# Prolonged Cardiopulmonary Bypass Time-Induced Endothelial Dysfunction via Glypican-1 Shedding, Inflammation, and Matrix Metalloproteinase 9 in Patients Undergoing Cardiac Surgery

**DOI:** 10.3390/biomedicines13010033

**Published:** 2024-12-27

**Authors:** Shiyi Li, Katherine V. Nordick, Iván Murrieta-Álvarez, Randall P. Kirby, Rishav Bhattacharya, Ismael Garcia, Camila Hochman-Mendez, Todd K. Rosengart, Kenneth K. Liao, Nandan K. Mondal

**Affiliations:** 1 Michael E. DeBakey Department of Surgery, Division of Cardiothoracic Transplantation and Circulatory Support, Baylor College of Medicine, Houston, TX 77030, USA; 2 Department of Regenerative Medicine Research, Texas Heart Institute, Houston, TX 77030, USA; 3 Michael E. DeBakey Department of Surgery, Division of Cardiothoracic Surgery, Baylor College of Medicine, Houston, TX 77030, USA

**Keywords:** cardiopulmonary bypass, glypican-1, endothelial function, endothelial glycocalyx, cytokine, matrix metalloproteinase

## Abstract

Objectives: A prolonged cardiopulmonary bypass (CPB) time of over 180 min is linked to poorer outcomes and higher mortality in cardiac surgery. This study examines how glypican-1 shedding, matrix metallopeptidase 9 (MMP9), and the pro-inflammatory cytokine IL-1β may contribute to endothelial dysfunction in patients undergoing on-pump surgery with an extended CPB. Methods: Fifty-one patients undergoing cardiac surgical procedures were divided into two groups based on the intraoperative CPB duration: (i) normal CPB (<180 min, *n* = 23) and (ii) prolonged CPB (>180 min, *n* = 28). The preoperative, intraoperative, and postoperative plasma levels of glypican-1, MMP9, and IL-1β were measured. Results: Before surgery, the plasma levels of glypican-1, MMP9, and IL-1β were comparable between the normal CPB and the prolonged CPB groups. However, after the end of the CPB, all three markers showed significant elevation in the prolonged CPB group compared to the normal CPB group. Significant correlations were observed between the intraoperative and postoperative levels of MMP9, IL-1β, and glypican-1. A strong positive correlation was also observed between the intraoperative and postoperative levels of glypican-1 and the duration of the CPB. Conclusions: A prolonged CPB triggers a systemic inflammatory response and activates MMP9, leading to glypican-1 shedding and endothelial dysfunction.

## 1. Introduction

A cardiopulmonary bypass (CPB) is commonly used for cardiac surgical procedures. A prolonged CPB is linked to increased postoperative morbidity and mortality [1]. The exposure of blood to abnormal shear stress and contact with the artificial surfaces in the bypass circuit leads to the activation of pro-inflammatory cytokines, matrix metalloproteinases (MMPs), and endothelial dysfunction [2,3,4]. Understanding the intricate interactions between these substances and their effects on physiological processes during a CPB is important for developing therapies to prevent and treat adverse clinical complications related to CPB.

Endothelial injury occurring during a CPB in cardiac surgery is a major contributing factor in the development of postoperative complications [5]. Endothelial cells are coated by the extracellular surface glycocalyx (GCX), which is important to maintain vascular homeostasis by preserving barrier function, suppressing inflammation, and mediating flow-induced nitric oxide (NO) release. Glypican-1 and syndecan-1 are the core proteins in the GCX [6]. Syndecan-1, as a transmembrane proteoglycan, has been reported to shed during prolonged CPB, while glypican-1, as a glycosylphosphatidylinositol-anchored protein, is more sensitive to shear stress [7,8,9]. Glypican-1 is integral to endothelial nitric oxide synthase (eNOS) activation and NO production under flow, suggesting that its shedding may directly impair endothelial function under prolonged CPB stress [10,11]. However, the effect of a prolonged CPB on glypican-1 is poorly understood.

CPB initiation during cardiac surgery can increase the circulating levels of MMPs and pro-inflammatory cytokines [2,12]. Pro-inflammatory cytokines were reported to mediate MMP activation and upregulation [12,13,14,15]. Moreover, MMP activation can mediate GCX shedding [16]. The released GCX component can activate dendritic cells and secrete pro-inflammatory cytokines, further aggravating the shedding of GCX and impairing endothelial function. In this study, we hypothesized that a prolonged CPB could trigger a stronger inflammatory response and lead to the activation and upregulation of MMP9. This, in turn, may result in the shedding of glypican-1 and degradation of the GCX. We investigated the effects of prolonged CPB on glypican-1 and studied the interactions among MMP, pro-inflammatory cytokines, and glypican-1 in cardiac surgery patients.

## 2. Materials and Methods

### 2.1. Patient Enrollment, Clinical Parameters, and Sample Collection

A total of 51 patients scheduled for a coronary artery bypass graft (CABG), valve, or aortic surgical procedures utilizing CPB were enrolled in this study. Based on previous research, a prolonged CPB in cardiac surgery is defined as lasting over 180 min [1]. Among the participants, 23 patients experienced a CPB duration of less than 180 min (average: 123 min) and were classified as the normal CPB group (*n* = 23). The remaining 28 patients experienced a CPB duration exceeding 180 min (average: 233 min) and were placed in the prolonged CPB group (*n* = 28). Exclusion criteria included the following: age under 18, emergency cardiac surgery, active myocarditis, hypertrophic cardiomyopathy, severe pulmonary hypertension, significant ventricular arrhythmias, and known malignancies. Baseline blood samples (pre-operative: Pre-OP) were collected 1 to 3 days before or on the day of surgery. Intra-operative samples (Intra-OP) were obtained by the cardiovascular anesthesia team once the patients were off the CPB. Post-operative samples (Post-OP) were collected at discharge. The venous blood samples were drawn into BD Vacutainer^®^ blood collection tubes (BD, Franklin Lakes, NJ, USA). Plasma was separated by centrifugation at 2500 rpm for 15 min (using an LC-8 series centrifuge, Benchmark Scientific, Sayreville, NJ, USA) and stored in 1.5 mL Eppendorf tubes at −80 °C for future analysis. Electronic medical chart reviews were performed to obtain clinical variables, including patient demographic characteristics, prior disease history, surgical procedures, Intensive Care Unit (ICU) stays, postoperative complications, hospital stays, and routine laboratory information.

### 2.2. Biochemical Estimation of Plasma Glypican1, MMP9, and IL-1β

The plasma levels of glypican-1, matrix metalloproteinase-9 (MMP9), and interleukin-1 beta (IL-1β) were assessed using commercially available Enzyme-Linked Immunosorbent Assay (ELISA) kits. Specifically, the kits used were ELH-GPC1-Glypican1 for glypican-1 (Ray Biotech, Peachtree Corners, GA, USA), KE00164-MMP9 for MMP9 (ProteinTech, Rosemont, IL, USA), and KE00021-IL-1β for IL-1β (ProteinTech, Rosemont, IL, USA). The assays were performed according to the manufacturer’s instructions to ensure the accuracy and reliability of the results. Proper controls and calibrators were utilized as specified to validate the findings.

### 2.3. Statistical Analysis

Data analyses and graphical presentations were carried out using GraphPad Prism version 10.3 (GraphPad Software, Inc., La Jolla, CA, USA) and SAS software version 9.4 (SAS Institute, Cary, NC, USA). Statistical comparisons between the groups were performed using a non-parametric one-way analysis of variance (ANOVA) with the Kruskal–Wallis test for the continuous variables, which are reported as the median with the interquartile range (IQR), and the Fisher’s exact test for the categorical variables (*n*, %). The experimental data are presented as the median with the IQR. Univariate analysis was conducted using the Spearman’s rank correlation test to assess the relationship between two measurable continuous variables, with the results expressed as rho values. A probability level of *p* < 0.05 was considered statistically significant.

## 3. Results

### 3.1. Baseline Demographics and Clinical Characteristics in Normal vs. Prolonged CPB Patients

Table 1 summarizes the baseline demographic data and the patient characteristics. All parameters, including the types of cardiac surgical procedures performed, were comparable between the normal CPB group and the prolonged CPB group. Most patients in the study were white and overweight, and a significant number had a history of smoking, alcohol abuse, and hypertension. In the normal CPB group, the baseline systolic and diastolic blood pressures were relatively higher than those in the prolonged CPB group.

### 3.2. Surgical Parameters and Outcomes in Normal vs. Prolonged CPB Patients

The intraoperative and postoperative characteristics are summarized in Table 2. Compared to patients in the normal CPB group, the median lengths of the CPB and aortic clamp were 1.9 and 1.8 times greater in the prolonged CPB group. The median length of surgery was around 5 h in a normal CPB, while it was approximately 7 h in the prolonged CPB group. The incidence of open-chest cardiac surgeries and the subsequent length of ICU stays were higher in the prolonged CPB group compared to the normal CPB group, although those differences were not statistically significant between our study groups. The ICU mortality differed notably between the groups, with six patients in the prolonged CPB group passing away during their ICU stay, while no mortality was reported in the normal CPB group. Moreover, the mechanical ventilation time was over 2-fold higher in the prolonged CPB group compared to the normal CPB group. In the 1-month postoperative outcomes, patients in the prolonged CPB group experienced higher rates of complications, including arrhythmia, acute kidney injury (AKI), and infections. Notably, the incidence of AKI was significantly higher in the prolonged CPB group compared to the normal CPB group.

### 3.3. Laboratory Hematology and Blood Chemistry in the Normal vs. Prolonged CPB Patients

The routine laboratory profiles for the normal CPB group patients and the prolonged CPB group patients are summarized in Table 3. Before the surgery, all the parameters were comparable between the normal CPB group and the prolonged CPB group. However, during the surgery, we noticed that the prolonged CPB group patients had significantly higher levels of creatinine and a significantly lower estimated glomerular filtration rate (eGFR) than the normal CPB group. Moreover, the prolonged CPB group patients still showed a significantly lower eGFR and relatively higher creatinine levels at discharge. The postoperative level of mean corpuscular hemoglobin (MCH) was also lower in the prolonged CPB group.

### 3.4. Temporal Changes in Plasma Glypican-1, MMP9 and IL-1β in Normal vs. Prolonged CPB Patients

**Figure 1:**
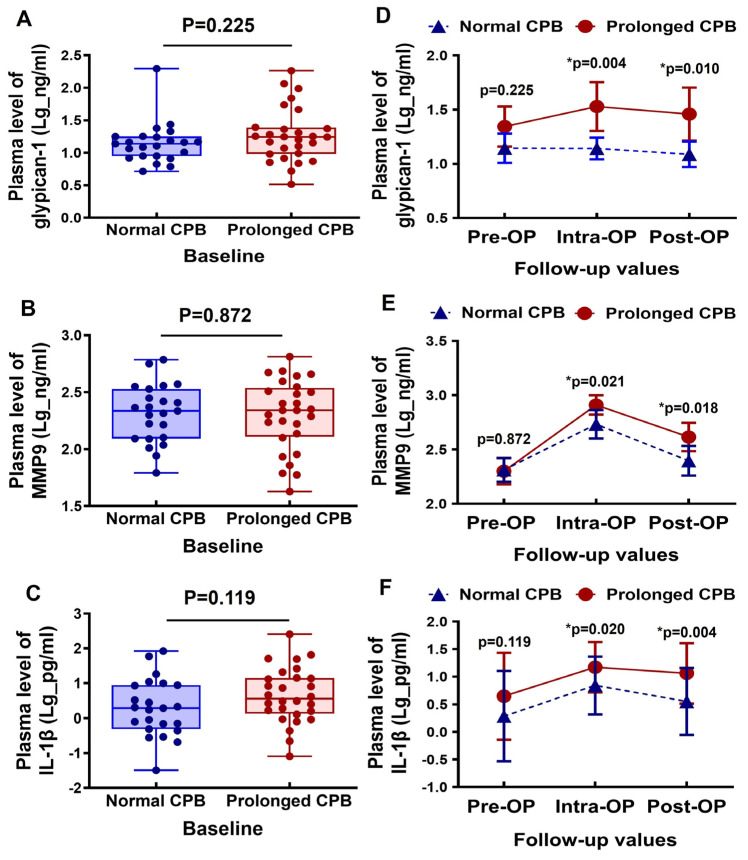
Changes in the circulating glypican-1, MMP9, and IL-1β levels in cardiac surgery patients with a normal CPB and prolonged CPB duration. (**A**–**C**) Box-and-whisker plots showing the preoperative (baseline) levels of (**A**) glypican-1, (**B**) MMP9, and (**C**) IL-1β between the normal CPB and prolonged CPB group patients. The lines across each box represent the median value. The lines that extend from the top and bottom of each box represent the lowest and highest observations still inside the lower and upper limits of confidence. (**D**–**F**) Line arts showing the change in the dynamic pre- and post-cardiac surgery timepoints of (**D**) glypican-1, (**E**) MMP9, and (**F**) IL-1β plasma level between the groups. * *p* < 0.05 is considered significant.

The baseline plasma levels of glypican-1, MMP9, and IL-1β were comparable between the normal CPB and the prolonged CPB groups (Figure 1A–C). During the intraoperative period, the glypican-1 levels slightly increased in the patients undergoing a prolonged CPB, followed by a slight decrease in the postoperative period. In contrast, the glypican-1 levels remained generally stable in the patients with a normal CPB duration. Additionally, the circulating glypican-1 levels were significantly higher in the prolonged CPB group during both the intraoperative and postoperative stages compared to the normal CPB group (Figure 1D). The circulating MMP9 levels significantly increased during the intraoperative period in both groups, but they returned to baseline levels in the normal CPB group during the postoperative period. In the prolonged CPB group, however, the postoperative MMP9 levels remained relatively elevated (see Figure 1E). Notably, the MMP9 levels were significantly higher in the prolonged CPB group at both the intraoperative and postoperative stages compared to the normal CPB group (Figure 1E). The inflammatory biomarker IL-1β was elevated during the intraoperative period and decreased postoperatively; however, it remained significantly higher in the prolonged CPB group during both stages compared to the normal CPB group (Figure 1F).

### 3.5. Relationship Between the Plasma Markers During and After the Surgery

**Figure 2:**
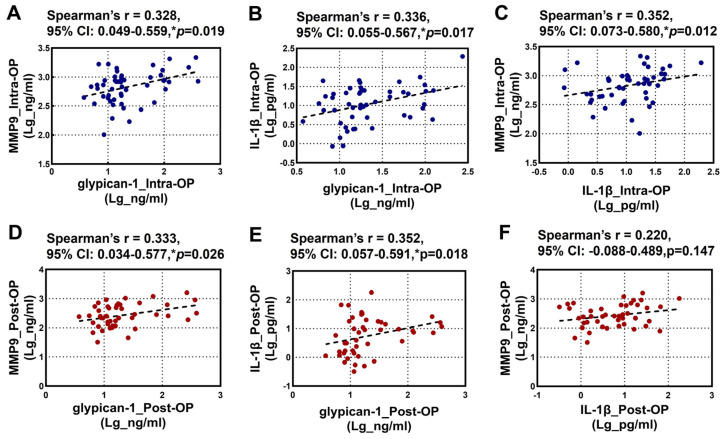
Spearman’s rank correlation analysis for the Intra-OP and Post-OP circulating glypican-1, MMP9, and IL-1β with each other. (**A**–**C**) Scatter plots showing the correlations between the intra-operative (**A**) glypican-1 and MMP9; (**B**) IL-1β and glypican-1, and (**C**) MMP9 and IL-1β; (**D**–**F**) Scatter plots showing the correlations between the post-operative (**D**) glypican-1 and MMP9, (**E**) IL-1β and glypican-1, and (**F**) MMP9 and IL-1β. Note—CI: confidence intervals; r: Spearman’s Rho. * *p* < 0.05 is considered significant.

The relationships between the intraoperative and postoperative plasma levels of glypican-1, MMP9, and IL-1β are illustrated in Figure 2. Spearman’s rank correlation analysis revealed a significant positive correlation among the intraoperative levels of glypican-1, MMP9, and IL-1β (Figure 2A–C). Additionally, a significant positive correlation was observed between the postoperative glypican-1 and MMP9 levels (Figure 2D). A similar positive correlation was noted between the postoperative glypican-1 and IL-1β levels (Figure 2E). However, no significant correlation was found between the postoperative MMP9 and IL-1β levels (Figure 2F).

### 3.6. Relationship Between the CPB Times and Plasma Markers During and After the Surgery

**Figure 3:**
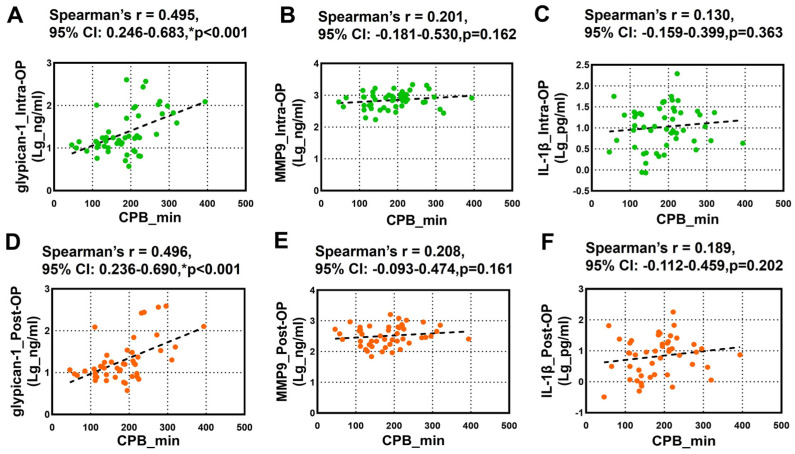
Spearman’s rank correlation analysis for the Intra-OP and Post-OP circulating glypican-1, MMP9, and IL-1β and the CPB duration. (**A**–**C**) Scatter plots showing the correlation between the CPB duration and the intraoperative (**A**) glypican-1, (**B**) MMP9, and (**C**) IL-1β; (**D**–**F**) Scatter plots showing the correlation between the CPB duration and the postoperative (**D**) glypican-1, (**E**) MMP9, and (**F**) IL-1β. Note–CI: confidence intervals; r: Spearman’s Rho. * *p* < 0.05 is considered significant.

The relationship between the CPB duration and the circulating glypican-1, MMP9, and IL-1β was further tested using Spearman’s rank correlation analysis. We noticed a robust positive correlation between the overall intra-OP and post-OP circulating glypican-1 and the CPB duration (Figure 3A,D). However, we did not observe any significant correlation between the intra-OP and post-OP circulating MMP9 (Figure 3B,E) and IL-1β (Figure 3C,F) and the CPB duration.

## 4. Discussion

A CPB temporarily takes over the functions of the heart and the lungs during surgical procedures. It maintains blood flow to the body and organs while providing a clear surgical field for surgeons. However, many studies have shown that the prolonged use of a CPB—lasting more than 180 min—can lead to poorer postoperative outcomes and increased mortality rates [1,17,18]. Our study discovered that a prolonged CPB time triggered a significant systemic inflammatory response and MMP9 activation, leading to glypican-1 shedding. This shedding may contribute to endothelial dysfunction, resulting in poorer postoperative outcomes. Glypican-1 shedding could be a potential target for alleviating endothelial dysfunction and improving clinical outcomes in cardiac surgery patients. We also noticed that an extended CPB duration is associated with higher rates of postoperative AKIs and increased mortality rates following cardiac surgery. A prior multicenter observational study examined the associations between perioperative characteristics, intraoperative and postoperative factors, and mortality and morbidity in open-heart surgery patients and found that a cardiopulmonary bypass duration exceeding 180 min was the strongest factor associated with a postoperative AKI [18]. Moreover, several retrospective observational studies have shown that patients who developed a postoperative AKI experienced longer CPB durations during cardiac surgery [19,20]. A longer CPB duration has been recognized as an independent risk factor for postoperative AKIs during cardiac surgery. In this study, we observed consistent results that the prolonged CPB group patients had significantly lower intraoperative and postoperative eGFR and significantly higher rates of postoperative AKIs. Endothelial dysfunction plays a vital role in the development of AKIs, and endothelial cells are the first cells to sense changes caused by ischemia that lead to an AKI [21]. Similarly, the increased degradation of the endothelial GCX in pediatric open-heart surgery following a CPB can significantly raise the risk of metabolic acidosis and renal dysfunction [22]. Glypican-1 is one of the major components of endothelial GCX, which works as an endothelial barrier and maintains endothelial integrity. Increased shedding of glypican-1 indicates the degradation of GCX, which may impair endothelial function. A prior study reported that glypican-1 expression depends on the duration of shear stress; glypican-1 can mediate the endothelial nitric oxide synthase (NOS) activation under shear stress, thereby protecting the endothelial function [10]. Moreover, glypican-1 is one of the key factors responsible for shear-induced endothelial NOS activation and NO production [8,23]. The depletion of NO during CPB can lead to worse postoperative outcomes, especially for postoperative AKIs [24]. People show great interest in NO supplementation during CPB to reduce postoperative AKIs in cardiac surgery. One recent randomized trial revealed that NO supplementation in patients undergoing cardiac surgery with CPB can significantly reduce the rates of postoperative AKI [25]. Our study may provide further insight into the use of NO supplementation during cardiac surgery with CPB to reduce the incidence of postoperative AKIs. Furthermore, it suggests that glypican-1 could be a potential therapeutic target to protect endothelial function and optimize perioperative management in patients undergoing prolonged CPB during cardiac surgery. On the other hand, recent evidence has highlighted the protective effects of sodium-glucose cotransporter-2 (SGLT2) inhibitors on endothelial function [26]. In both in vitro and in vivo studies, SGLT2 inhibitors have demonstrated protective effects on endothelial function by enhancing eNOS activation and NO bioavailability [27,28,29]. Administering SGLT2 inhibitors to patients undergoing a prolonged CPB may offer promising benefits in mitigating prolonged CPB-induced endothelial dysfunction.

A study by Robich et al. reported that a prolonged CRB duration is associated with endothelial GCX shedding and degradation [7]. However, their study defined a prolonged CPB as a CPB duration exceeding 102 min, based on the median CPB time observed in their cohort. Another endothelial GCX marker (syndecan-1) was found to significantly upregulate in the prolonged CPB group; moreover, its level was found to be significantly positively correlated with the CPB time [7]. Glypican-1 and syndecan-1 are the core proteins in the GCX [6]. However, Glypicans are bound to the plasma membrane by glycosylphosphatidylinositol anchors, whereas syndecans are transmembrane; glypican-1 is more sensitive to shear stress than syndecan-1 [8,9]. Despite differences in the definition of a prolonged CPB, we both observed consistent results indicating that a prolonged CPB can impair endothelial function by promoting GCX shedding and degradation and that circulating soluble endothelial GCX markers may serve as useful indicators to assess CPB-related damage in cardiac surgery. On the other hand, endothelial GCX has been proposed as a potential therapeutic target to alleviate CPB-related endothelial damage [30,31]. In a previous study by Dekker et al., a CPB was shown to induce acute microcirculatory perfusion disturbances via prolonged endothelial GCX shedding, and on-pump cardiac surgery was associated with a prolonged impairment and delayed recovery of both microcirculatory perfusion and function [32].

MMPs are the key enzymes that are responsible for the degradation of the endothelial GCX [33,34]. MMP expression and activity are primarily regulated by oxidative stress and the inflammatory response [33,35]. High levels of oxidative stress and inflammation can upregulate the expression of MMPs, thereby increasing the GCX degradation. Oxidative stress and inflammation can be significantly upregulated during CPB [36,37]. Many studies have reported that MMP9 levels can be significantly upregulated after CPB [38,39]. In our study, we observed consistent results showing that the MMP9 levels significantly upregulated in both the groups of patients during the CPB, and its level returned to baseline level in the normal CPB group patients while remaining at a high level in the prolonged CPB group patients after the surgery. Gao et al. reported that MMP9 was an important contributor to GCX shedding during a CPB [40]. Blocking MMP9 can alleviate GCX shedding and restore the endothelial barrier function [41]. Interestingly, one recent study found that increasing MMP9 expression and endothelial GCX shedding can predict an extended ICU stay following cardiac surgery with a CPB [42]. In our study, we observed a significant positive correlation between MMP9 and glypican-1, suggesting that MMP9 may involve glypican-1 shedding, contributing to endothelial GCX degradation during and after cardiac surgery. On the other hand, pro-inflammatory cytokines, such as IL-1β, are known to induce the expression and activity of MMP9 [43,44]. Previous mechanistic studies on animal models have shown that downregulation of MMP-9 expression reduces endothelial GCX shedding both in vitro and in vivo [16]. Another previous study from Homer et al. reported that IL-1β can increase MMP9 expression and activity, thereby contributing to endothelial permeability [45]. In our study, we consistently observed a significant positive correlation between glypican-1, MMP9, and IL-1β both intraoperatively and postoperatively (although not preoperatively), suggesting a potential association between these molecules in contributing to GCX shedding after the patients experienced shear stress generated by the CPB used. This interplay among inflammation, MMP activation, and endothelial GCX degradation may help explain the underlying mechanisms of CPB-induced endothelial dysfunction and complications in cardiac surgery patients. As such, therapy that targets glypican-1 shedding may benefit cardiac surgery patients with a prolonged CPB by reducing post-operative complications and decreasing the mortality rate.

There are several limitations in our study: (i) The sample size is relatively small, and all cases are from a single-center experience. (ii) In this study, we only investigated one endothelial biomarker (glypican-1) and did not compare the findings with the other GCX marker (syndecan-1) to determine which one is better to assess CPB damage. Future studies should be performed to compare glypican-1 and syndecan-1 under the impact of CPB. (iii) A prolonged aortic clamp time and more complex surgical procedures are often seen in patients requiring an extended CPB, which can exacerbate systemic inflammation and oxidative stress, thereby promoting MMP9 activation and glypican-1 shedding. To account for these potential confounders in future studies, larger cohorts with subgroup analyses and multivariate regression models should be utilized to isolate the independent effect of the CPB duration on endothelial dysfunction.

## 5. Conclusions

A sustained CPB for over 180 min during cardiac surgery has been linked to significantly worse outcomes after surgery and a higher risk of mortality. This extended duration of the CPB may instigate a systemic inflammatory response, which activates MMP9. This activation contributes to the shedding of glypican-1, a key component that plays a role in maintaining endothelial function. When glypican-1 is shed, it leads to endothelial dysfunction, further complicating recovery. Further investigation focusing on the prevention of glypican-1 shedding could reduce the negative impacts associated with a prolonged CPB, leading to an improved recovery and better overall patient outcomes.

## Figures and Tables

**Table 1:** Demographic and baseline clinical characteristics of the normal CPB and prolonged CPB patients.

Characteristics	Normal CPB (N = 23)	Prolonged CPB (N = 28)	*p*-Value
Demography			
Age in years, Median (IQR)	68 (63–77)	65 (62–72)	0.59
Sex, *n* (% male)	12 (52.17%)	18 (64.29%)	0.41
Race			1.00
Caucasian white, *n* (%)	18 (78.26%)	22 (78.57%)	
African American, *n* (%)	2 (8.70%)	2 (7.14%)	
Asian, *n* (%)	3 (13.04%)	4 (14.29%)	
Height in meters, Median (IQR)	1.70 (1.60–1.80)	1.73 (1.67–1.79)	0.34
Weight in kilograms, Median (IQR)	82.00 (71.70–96.20)	91.27 (77.00–103.10)	0.24
BMI, kg/m^2^, Median (IQR)	28.14 (23.90–32.90)	29.74 (25.75–33.95)	0.33
BSA, m^2^, Median (IQR)	1.93 (1.80–2.08)	2.06 (1.85–2.17)	0.20
History of smoking, *n* (%)	10 (43.48%)	14 (50.00%)	0.78
History of alcohol abuse, *n* (%)	11 (47.83%)	16 (57.14%)	0.58
Hypertension, *n* (%)	19 (82.61%)	21 (75.00%)	0.73
Diabetes, *n* (%)	8 (34.78%)	7 (25.00%)	0.54
COPD, *n* (%)	2 (8.70%)	2 (7.14%)	1.00
ESRD, *n* (%)	2 (8.70%)	2 (7.14%)	1.00
Peripheral vascular disease, *n* (%)	1 (4.35%)	2 (7.14%)	1.00
Cerebral vascular accident, *n* (%)	4 (17.39%)	3 (10.71%)	0.69
Prior cardiac surgeries, *n* (%)	1 (4.55%)	2 (7.14%)	1.00
SBP (mmHg), Median (IQR)	138 (123–155)	129 (116–141)	0.09
DBP (mmHg), Median (IQR)	72 (68–79)	69 (64–73)	0.05
Echocardiographic parameters			
LviDd in centimeters, *n* (%)	4.61 (3.84–5.20)	4.91 (4.39–5.28)	0.35
LVEF (%)	56.20 (55.10–61.80)	57.66 (54.80–64.10)	0.97
Surgical Procedure			0.77
CABG, *n* (%)	3 (13.04%)	6 (21.43%)	
Valve, *n* (%)	16 (69.57%)	18 (64.29%)	
CABG with Valve, *n* (%)	4 (17.39%)	4 (14.29%)	

Note: The continuous variables are presented as the median with the interquartile range (IQR), while the categorical variables are presented as the number (*n*) and percentage (%). Statistical analysis comparing the groups was performed using a non-parametric one-way ANOVA with the Kruskal–Wallis Test for the continuous variables and the Chi-square test for the categorical variables. *p* < 0.05 is considered statistically significant. CPB: cardiopulmonary bypass; BMI: body mass index; BSA: body surface area; COPD: chronic obstructive pulmonary disease; ESRD: End-stage renal disease; SBP: systolic blood pressure; DBP: diastolic blood pressure; LviDd: left ventricular internal diameter at end-diastole; LVEF: left ventricular ejection fraction; CABG: coronary artery bypass graft; Valve: Aortic/mitral replacement or repair.

**Table 2:** Surgery parameters and outcomes of the normal CPB and prolonged CPB patients.

Characteristics	Normal CPB (N = 23)	Prolonged CPB (N = 28)	*p*-Value
Length of CPB in minutes, Median (IQR)	124 (111–142)	235 (201–270)	<0.01 *
Length of aortic clamp in minutes, Median (IQR)	87 (74–107)	155 (120–181)	<0.01 *
Length of surgery in minutes, Median (IQR)	278 (221–308)	430 (350–468)	<0.01 *
Sternotomy, *n* (%)	12 (52.17%)	20 (71.43%)	0.25
ICU mortality, *n* (%)	0	6 (21.43%)	0.03 *
ICU stay in days, Median (IQR)	7 (2–10)	19 (3–17)	0.22
Mechanical ventilation time in hours, Median (IQR)	21 (3–7)	54 (6–20)	0.02 *
Arrhythmia, *n* (%)	5 (21.74%)	12 (42.86%)	0.14
AKI, *n* (%)	1 (4.35%)	8 (28.57%)	0.03 *
Infection, *n* (%)	1 (4.35%)	4 (14.29%)	0.36

Note: The continuous variables are presented as the median with the interquartile range (IQR), while the categorical variables are presented as the number (*n*) and percentage (%). Statistical analysis comparing the groups was performed using a non-parametric one-way ANOVA with the Kruskal–Wallis Test for the continuous variables and the Chi-square test for the categorical variables. * *p* < 0.05 is considered statistically significant. CPB: cardiopulmonary bypass; ICU: intensive care unit; AKI: acute kidney injury.

**Table 3:** Routine laboratory hematology and blood chemistry parameters of the normal CPB and prolonged CPB patients.

Characteristics	Normal CPB (N = 23)	Prolonged CPB (N = 28)	*p*-Value
Preoperative laboratory parameters			
Leukocytes (10^3^/μL), Median (IQR)	7.17 (5.40–8.50)	7.38 (5.10–8.20)	0.91
Erythrocyte (10^3^/μL), Median (IQR)	4.26 (4.03–4.43)	4.15 (3.68–4.68)	0.79
Hemoglobin (g/dL), Median (IQR)	12.71 (11.60–14.50)	11.69 (10.30–13.30)	0.08
Hematocrit (%), Median (IQR)	38.55 (34.40–42.60)	38.26 (33.00–41.10)	0.29
MCH (pg), Median (IQR)	29.83 (28.80–31.00)	28.27 (27.70–30.15)	0.09
Platelets (10^3^/μL), Median (IQR)	235 (188–263)	211 (166–259)	0.29
Creatinine (mg/dL), Median (IQR)	1.08 (0.77–1.15)	1.13 (0.90–1.23)	0.17
eGFR (ml/min/1.73 m^2^), Median (IQR)	74.57 (69.00–86.00)	70.11 (63.50–83.00)	0.16
INR, Median (IQR)	1.36 (1.10–1.36)	1.36 (1.10–1.36)	0.08
Intraoperative laboratory parameters			
Leukocytes (10^3^/μL), Median (IQR)	13.58 (9.70–17.85)	15.06 (10.00–19.60)	0.46
Erythrocyte (10^3^/μL), Median (IQR)	3.31 (2.80–3.82)	3.30 (2.91–3.68)	0.98
Hemoglobin (g/dL), Median (IQR)	10.02 (8.90–11.60)	9.42 (8.70–10.15)	0.15
Hematocrit (%), Median (IQR)	30.40 (26.90–33.70)	29.25 (26.95–30.90)	0.44
MCH (pg), Median (IQR)	30.37 (29.10–31.60)	28.74 (27.80–30.50)	0.08
Platelets (10^3^/μL), Median (IQR)	154 (107–172)	125 (86–148)	0.13
Creatinine (mg/dL), Median (IQR)	1.14 (0.69–0.95)	1.80 (0.84–1.16)	* <0.01
eGFR (ml/min/1.73 m^2^), Median (IQR)	82.78 (75.00–100.00)	66.46 (62.00–86.00)	* <0.01
INR, Median (IQR)	1.61 (1.39–1.71)	1.66 (1.46–1.83)	0.14
Postoperative laboratory parameters			
Leukocytes (10^3^/μL), Median (IQR)	10.73 (9.10–11.20)	10.38 (7.70–11.75)	0.46
Erythrocyte (10^3^/μL), Median (IQR)	3.17 (2.93–3.35)	3.11 (2.80–3.36)	0.50
Hemoglobin (g/dL), Median (IQR)	9.41 (8.40–9.90)	8.83 (8.15–9.15)	0.07
Hematocrit (%), Median (IQR)	28.58 (26.40–30.70)	27.44 (25.50–29.10)	0.20
MCH (pg), Median (IQR)	30.18 (28.60–31.50)	28.58 (28.05–30.10)	* 0.04
Platelets (10^3^/μL), Median (IQR)	162 (110–215)	126 (83–186)	0.13
Creatinine (mg/dL), Median (IQR)	0.94 (0.64–0.91)	1.12 (0.74–1.36)	0.05
eGFR (ml/min/1.73 m^2^), Median (IQR)	88.57 (84.00–102.00)	71.07 (45.00–96.00)	* 0.02
INR, Median (IQR)	1.43 (1.27–1.71)	1.53 (1.28–1.61)	0.46

Note: The continuous variables are presented as the median with the interquartile range (IQR), while the categorical variables are presented as the number (*n*) and percentage (%). Statistical analysis comparing the groups was performed using a non-parametric one-way ANOVA with the Kruskal–Wallis Test for the continuous variables and the Chi-square test for the categorical variables. * *p* < 0.05 is considered statistically significant. MCH: mean corpuscular hemoglobin; eGFR: estimated glomerular filtration rate; INR: international normalized ratio.

## Data Availability

The data presented in the study are available on request from the corresponding author(s).
